# Clinical potential of the Hippo-YAP pathway in bladder cancer

**DOI:** 10.3389/fonc.2022.925278

**Published:** 2022-07-15

**Authors:** Xin Cheng, Kecheng Lou, Liang Ding, Xiaofeng Zou, Ruohui Huang, Gang Xu, Junrong Zou, Guoxi Zhang

**Affiliations:** ^1^ First Clinical College, Gannan Medical University, Ganzhou, China; ^2^ Department of Urology, First Affiliated Hospital of Gannan Medical University, Ganzhou, China; ^3^ Institute of Urology, First Affiliated Hospital of Gannan Medical University, Ganzhou, China; ^4^ Department of Jiangxi Engineering Technology Research Center of Calculi Prevention, Gannan Medical University, Ganzhou, China

**Keywords:** bladder cancer, Hippo pathway, YAP, chemoresistant, cancer stem cell

## Abstract

Bladder cancer (BC) is one of the world’s most frequent cancers. Surgery coupled with adjuvant platinum-based chemotherapy is the current standard of therapy for BC. However, a high proportion of patients progressed to chemotherapy-resistant or even neoplasm recurrence. Hence, identifying novel treatment targets is critical for clinical treatment. Current studies indicated that the Hippo-YAP pathway plays a crucial in regulating the survival of cancer stem cells (CSCs), which is related to the progression and reoccurrence of a variety of cancers. In this review, we summarize the evidence that Hippo-YAP mediates the occurrence, progression and chemotherapy resistance in BC, as well as the role of the Hippo-YAP pathway in regulating bladder cancer stem-like cells (BCSCs). Finally, the clinical potential of Hippo-YAP in the treatment of BC was prospected.

## Introduction

Worldwide, BC is the 11th most common malignancy, with more than 570,000 new cases and 210,000 deaths in 2020 (1), and the incidence is increasing (2). BC is divided into nonmuscle-invasive bladder cancer (NMIBC) and muscle-invasive bladder cancer (MIBC). NMBIC is less malignant, and the routine treatment is based on TURBT (transurethral resection of bladder tumor) combined with bladder perfusion chemotherapy or immunotherapy (3). MIBC is more aggressive, and the classical treatment is radical cystectomy combined with platinum-based chemotherapeutic (4). The preferred treatment for metastatic MIBC is platinum-based chemotherapy. In cisplatin-ineligible patients, immunotherapy is preferred for PD-L1-positive patients, and carboplatin is chosen as an alternative therapy for PD-L1-negative patients (4, 5). Unfortunately, even with the tremendous efforts of current research on BC, the mortality rate of BC patients is still high (1). The most important factor affecting the prognosis of BC patients is that a large proportion of patients relapse after the first treatment for BC and are resistant to existing treatment regimens (6), with no effective therapeutic target to date (7, 8). Therefore, it is necessary to further investigate the mechanisms of BC pathogenesis, recurrence and drug resistance, and to screen effective targeted drugs for the treatment of advanced metastatic BC.

**Figure 1:**
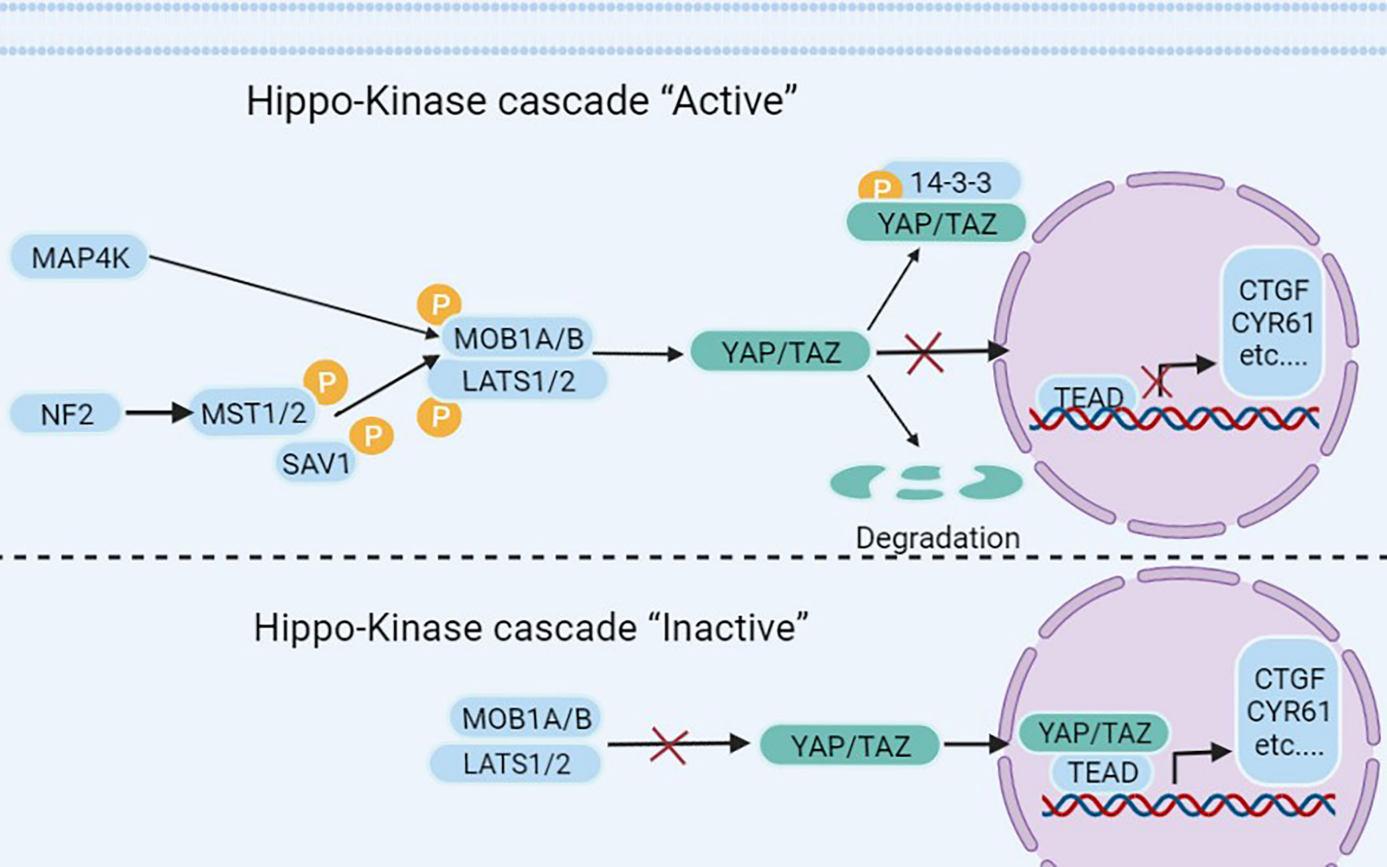
The Hippo pathway’s upstream serine-threonine kinase cascade regulates YAP/TAZ. MST1/2 and MAP4K families are the main kinases of the Hippo- kinases cascade. When they are phosphorylated, which subsequently inhibits the transcriptional activity of YAP (20) and TAZ (21) through phosphorylating LAST1/2 (22–25). On the contrary, when the Hippo-kinase cascade is “inactive”, it leads to YAP dephosphorylation, which translocates to the nucleus and binds to TEAD1–TEAD4, following with the transcription of downstream genes (26–28). Such as multiple anti-apoptotic and proliferative genes, including CTGF (connective tissue growth factor) and CYR61 (cysteine-rich angiogenic factor) (26–28). Other molecules regulating YAP/TAZ phosphorylation have also been reported in the literature, such as NDR1/2 (Nuclear Dbf2-related 1/2) (29), SRC (30–33), NLK (Nemo-like kinase) (34, 35), AMPK (5’adenosine monophosphate-activated protein kinase) (36–38), and JNK (c-Jun N-terminal kinase) (39) have all been found to directly phosphorylate and hence control YAP/TAZ. Finally, YAP/TAZ is regulated in a kinase-independent manner (18, 19, 40).

The Hippo-YAP signaling pathway plays a key role in stem cells and cancer cells (9, 10). The Hippo pathway, first identified in Drosophila melanogaster, has a role in regulating organ size (11) and is conserved in a variety of species, including humans (12). It is an important regulator of organ development, cell proliferation, dynamic balance, and regeneration (10, 13). Extracellular matrix, nutrition, cell density, cell polarity, mechanical transduction, and G protein-coupled receptors are all factors that regulate the Hippo-YAP pathway (14–17). The cytoplasmic kinase cascade and the nuclear transcription module are the two primary components of the Hippo-YAP pathway. The Hippo-kinase cascade is mainly composed of MAP4K, MST1/2, and LATS1/2 (18, 19). The nuclear transcriptional module of the Hippo pathway is a transcriptionally active motif with oncogenic effects composed of YAP (yes-associate protein), TAZ (transcriptional co-activators with PDZ binding sequences), and TEAD-1 (TEA domain family member 1), which are mainly regulated by the Hippo-kinase cascade (**Figure 1**). YAP/TAZ has a dominant role in numerous solid tumors (9, 13, 17, 41, 42), and increasing significance of elevated YAP/TAZ activity in BC (43).

In this review, we summarized the evidence that YAP would be a promising therapeutic target, regarding the association of YAP with BC onset, progression, postoperative recurrence, chemoresistance, and metastasis. In addition, we emphasized the role of the Hippo-YAP pathway in regulating BCSCs (bladder cancer stem-like cells), as well as the hitherto unanswered question that how the nuclear transcriptional module of the Hippo pathway is over-activated in BC. At last, the clinical potential and pharmacology direction of Hippo-YAP were discussed in this paper.

## Aberrant activation of YAP/TAZ in BC

The role of YAP in BC has received increasing attention, and many studies have shown that YAP is a clinical marker of BC progression (44) and a key molecule contributing to postoperative recurrence and chemotherapy resistance in BC (45). Levels of YAP correlate positively with pathological grade of BC (46), and enhanced YAP activity has been shown in the majority of solid tumors (42), including lung, liver, sarcoma, pancreas, and breast (9, 10, 41).

**Figure 2:**
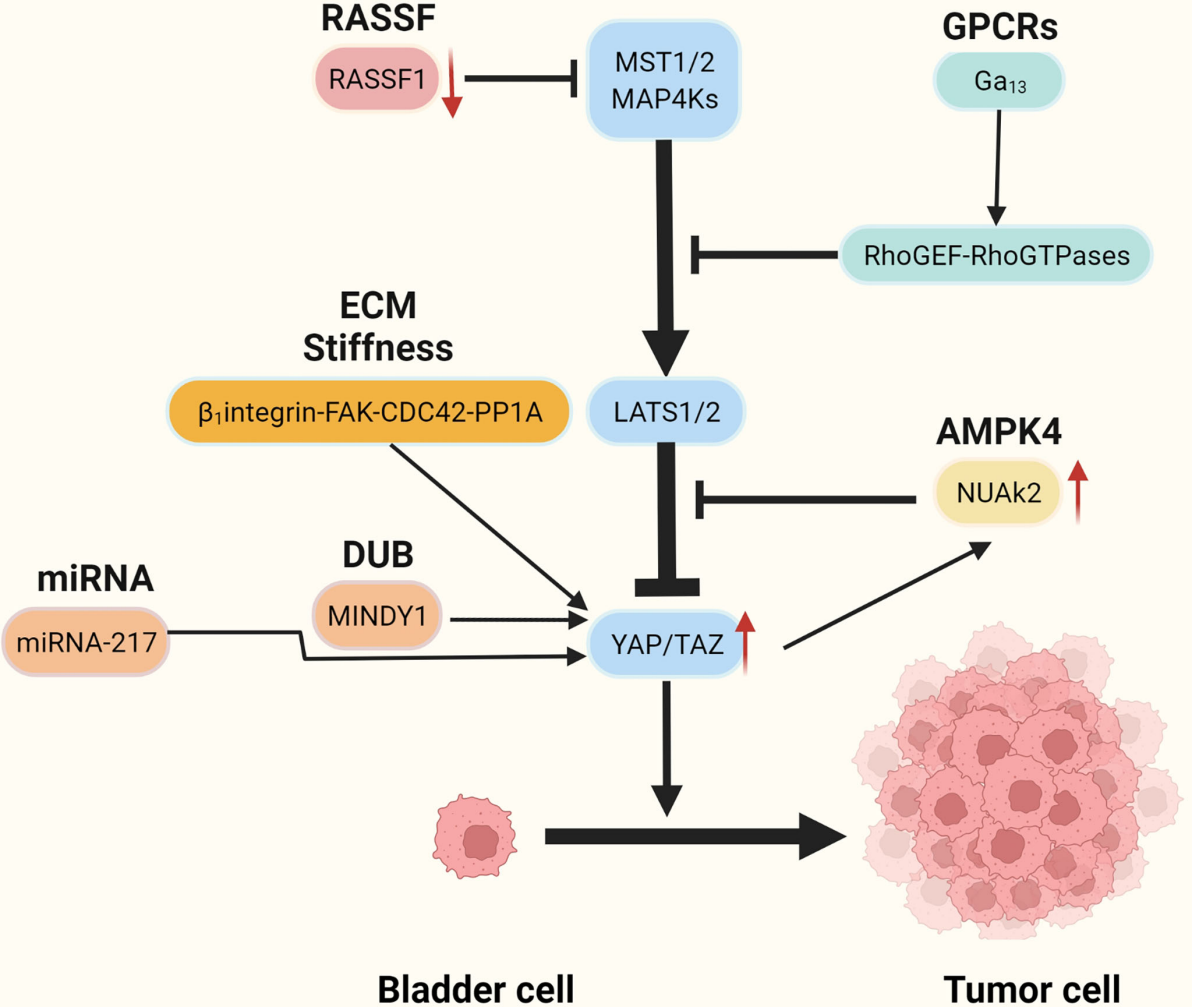
The mechanism of aberrant activation of YAP/TAZ.

Recent studies have reported that YAP is highly expressed in BC tissues and that knockdown of the YAP gene impaired the proliferation and migratory capacity of BC cells (47). High YAP expression correlates with poor prognosis in patients with BC (48). It is not clear how YAP becomes overactivated and forces BC initiation and progress, but several possible mechanisms have recently been identified (**Figure 2** and **Table 1**).

**Table 1:** Functions of genes associated with the Hippo-YAP pathway.

Gene	Function of Hippo-YAP	Function of BC	Refere-nce
ALDH1	Activated YAP/TAZ	Progression and chemotherapy resistance	(49)
CDC42	Activated YAP/TAZ	Progression and recurrence	(45)
FAK	Activated YAP/TAZ	Progression and recurrence	(45)
FOXM1	Activated YAP/TAZ	Progression and chemotherapy resistance	(50)
GNA13	Activated YAP/TAZ	Progression	(51)
ITGB1	Activated YAP/TAZ	Progression and recurrence	(45)
LATS1/2	Inactivated YAP/TAZ	Inhibition	(18, 19)
MINDY1	Activated YAP/TAZ	Progression	(52)
MST1/2	Inactivated YAP/TAZ	Inhibition	(18, 19)
miRNA-217	Activated YAP/TAZ	Progression	(53)
NUAK2	Inactivated LATS1/2	Progression	(46)
NRF2	Activated YAP/TAZ	Progression and chemotherapy resistance	(50)
RhoA/B/C	Activated YAP/TAZ	Progression	(51)
RASSF1	Inactivated MAST1/2	Progression	(54)
PDGFB	Activated YAP/TAZ	Progression and chemotherapy resistance	(55)
PP1A	Activated YAP/TAZ	Progression and recurrence	(45)

### Mutant GNA13 gene activates YAP/TAZ

Heterotrimeric G-proteins are important signal transduction molecules triggered by a large class of GPCRs (G-protein-coupled receptors) (56). Dysregulation of the GPCRs-G-protein pathway in cancer has been reported to be very common (57–59). G-protein family mutations were related to several malignancies, such as GNAQ or GNA11 (G_q/11_ family)mutations are found in 90% of uveal melanomas (60, 61), 70% of pancreatic ductal carcinomas present GNAS (G_s_ family) mutations (62, 63), and 24% of epithelial T-cell lymphomas (64) GNAI2 (G_i/o_ family)mutation. *In vitro*, tumorigenic experiments found that the G_i/o_ family, G_q/11_ family, and G_12/13_ (GNA12 and GNA13) family mutation can promote oncogenic transformation (65–70).

**Figure 3:**
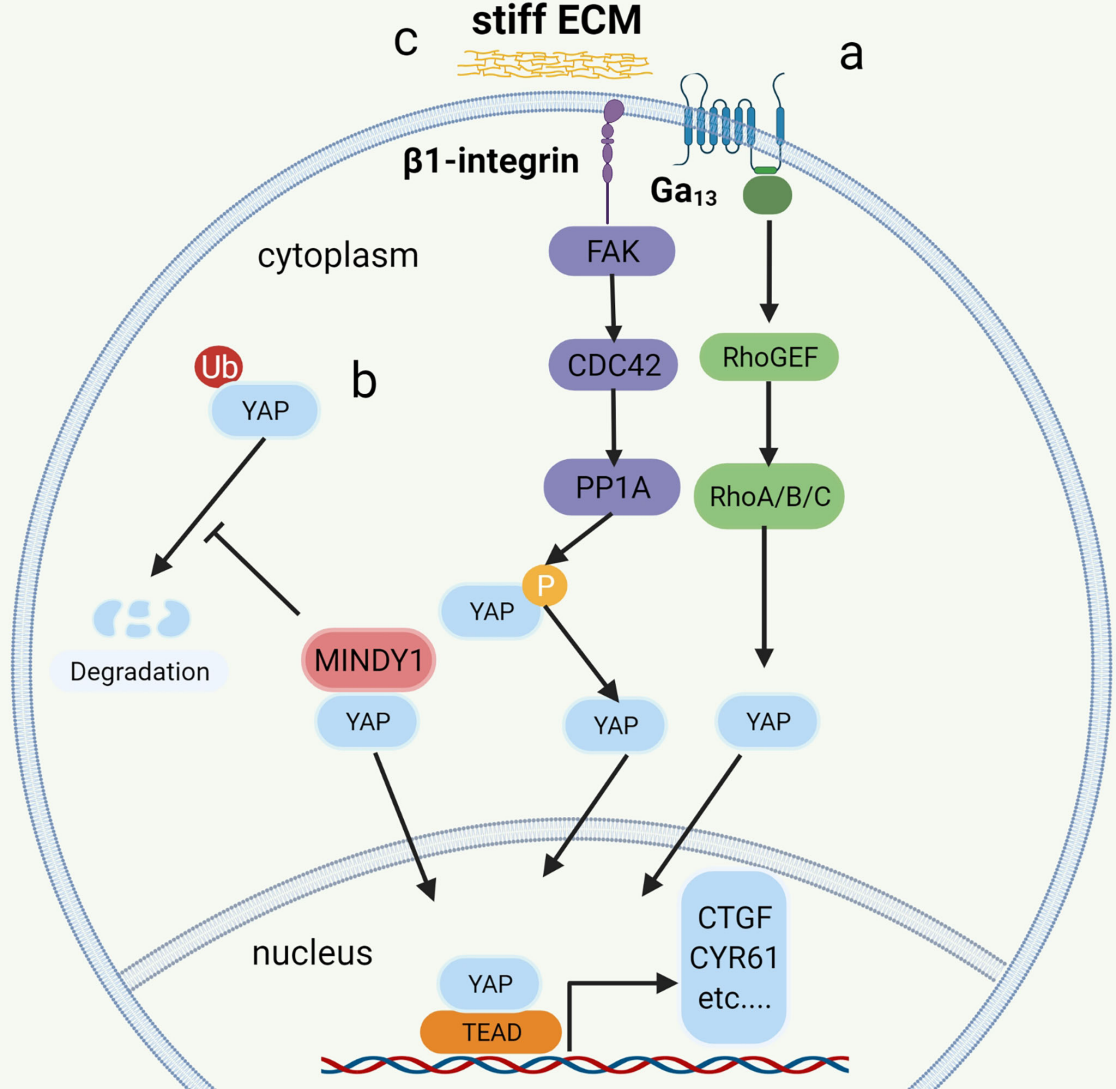
The mechanism of YAP regulation in bladder cancer. a: Mutation of G_12/13_ can significantly increase the transcriptional activity of YAP/TAZ by upregulating the RhoGEF-Rho GTPase cascade. b: MINDTY1 increases its stability and avoids degradation by removing the ubiquitin chain from YAP. c: ECM stiffness increases the nuclear localization of YAP by activating the integrin-FAK-CDC42-PP1A signaling pathway to dephosphorylate YAP.

Recent research based on bioinformatics analysis has shown that GNA13 mutation may be an oncogene in BC (59, 71, 72) and that the mutated GNA13 gene produces oncogenic effects by activating YAP/TAZ (51). This was confirmed by research by Dr. Maziarz, who showed that the Arg-200 mutation of GNA13 in BC can significantly increase YAP/TAZ transcriptional activity by upregulating the RhoGEF-Rho GTPase cascade in TCGA database and cellular experiments (51)(**Figure 3A**). *In vitro*, tumorigenic experiments showed that the GNA13Arg-200 mutant induced cancerization of cells (control group of unmutated cell lines) (51). Dr. Maziarz’s findings back up the theory that GNA13 hotspot mutations are a potential cause of BC, and that pharmacological inhibition of the Hippo-YAP pathway might be a feasible treatment option (51). This conclusion should be taken with a grain of salt because Dr. Maziarz’s experiment lacks clinical validation in multiple data centers and *in vivo* tumorigenic assays.

### NUAK2-LAST-YAP/TAZ positive feedback regulation loop

NUAK2 is a member of the AMPK kinase family, which has been extensively examined for its regulation of the Hippo-YAP pathway by regulating the Hippo kinase cassette (36–38, 73–76). Recent studies have shown that NUAK2 activity is significantly associated with aggressive, high-grade BC. Separate extracts of tumor cells from patients with high-grade and low-grade BC were tested and showed that NUAK2 expression in tumor cells was significantly higher in high-grade patients than in low-grade patients. Knockdown of NUAK2 gene in various cancer cell lines such as BC cell lines (TCCSUP, T24), colon cancer cell lines (SW480) and breast cancer cell lines (MDA-MB231 and MDA-MB468) significantly inhibited the transcriptional activity of YAP/TAZ and the proliferation ability of cancer cells (46). Further experiments revealed that the expression of NUAK2 was positively related to YAP/TAZ activity and negatively correlated with LAST activity. The regulatory effect of NUAK2 on YAP/TAZ was significantly diminished when LAST was knocked down, and the knockdown of YAP/TAZ decreased the expression of NUAK2. The Above research suggests the existence of a NUAK2-LAST-YAP/TAZ positive feedback regulatory loop in BCs with high activity of NUAK2 (46) (**Figure 2**).

### The ubiquitin-protease system regulates the Hippo-YAP

The ubiquitin proteasomes system (UPS) is a protein degradation pathway that exists in all eukaryotic cells. UPS is the most important regulated protein degradation system, which participates in the cell cycle process, cell survival, apoptosis, DNA repair, and antigen presentation (77). The imbalance of UPS can lead to increased or reduced degradation of key proteins that promote tumorigenesis (78). Recently, it has been reported that several ubiquitin-protein ligases (E3) in UPS, such as PRAJA1, ITCH, SIAH2, FBXW7, and WWP1, play an important role in regulating the expression of YAP. These enzymes can regulate the stability of YAP protein in cancer cells through ubiquitin and proteasome degradation (79, 80). The protein level of LATS kinase is controlled by E3 ubiquitin ligase-mediated degradation. In addition, LATS has a unique E3 chain, and MST1 also has its unique E3 ligase C-terminal recognition (81). The de-ubiquitin enzyme (DUB) is an enzyme with the opposite function of E3, such as MINDY1, which can increase its stability by removing the K48-linked ubiquitin chain from YAP. When it is exhausted, it can reduce the level of YAP protein and inhibit the YAP-TEAD-1 transcriptional activity, weakening the proliferation and invasiveness of cancer cells (52) (**Figure 3B**).

### ECM stiffness activates YAP

More and more studies have found that the extracellular matrix (ECM) determines the fate and behavior of cancer cells, including differentiation, proliferation, apoptosis, and migration (82). In addition to perlecan, fibrillary collagen, and laminin in ECM, overexpression of agrin leads to increased density of ECM and ECM stiffness (83), leading to abnormal signals activating integrin (mechanosensory receptor) and related pathways (83). It is reported that collagen stiffness in ECM promotes NMIBC to MIBC, which may also be one of the causes of postoperative BC recurrence (84). However, the function and role of the proteins in ECM and the related signal transduction pathways are still opaque. Fortunately, according to the latest research, it has been found that the integrin-FAK-CDC42-PP1A (45)signaling pathway leads to ECM stiffness to promote the progression and recurrence of BC (**Figure 3C**). In addition to the high expression of β1-integrin (encoded by ITGB1), FAK, and CDC42, high ECM stiffness is also associated with increased nuclear localization of YAP (45). Molecular docking data showed that integrin binds to FAK through hydrogen bonding (45). FAK activates CDC42-PP1A kinase and dephosphorylates YAP (85), thus increasing the nuclear localization of YAP (45).

### Other pathways related to YAP activation

RASSF1 is a tumor suppressor (86). Its inactivation leads to the occurrence and development of many kinds of tumors including BC (87, 88). Low expression of RASSF1 in BC is strongly associated with high expression of YAP, CTGF, and CYR61, in addition to high-risk BC (54). Further studies have found that decreased expression of RASSF1 in BC inactivated MST1/2, which leads to increased activity of the YAP-TEAD-1 and promotes the occurrence and development of BC (54)(**Figure 2**).

The role of exosomes as novel biological markers in tumorigenesis, progression, diagnosis, and treatment is being increasingly emphasized (89–91). The miRNA-217 is secreted through exosomes by BC mesenchymal cells (53), and miRNA-217 expression is significantly higher in BC cell lines than in normal human bladder cell lines (53). The miRNA-217 affects BC proliferation, migration, and apoptosis by regulating the transcription factor YAP and its target proteins CTGF, CYR61, and ANKRD1 (53) (**Figure 2**).

## Role of HIPPO-YAP pathway in BCSCS

### Role of BCSCs in BC

BCSCs are a subgroup of BC cells, which have stem-like properties such as high proliferation, self-renewal, and drug resistance (92). Progression, chemotherapy resistance, and heterogeneity of BC are significantly related to cancer stem-like cells (CSCs) (93–95). At present, the markers commonly used to identify BCSCs are CD44, CD133, ALDH1, OV6, BMI1, and ABCG2 (49, 55, 96, 97). Although, the specific mechanism of preserving the stem-like qualities of BCSCs remains unclear, encouragingly, several signaling pathways have recently been reported to regulate the proliferation, tumorigenesis, and chemoresistance of BCSCs, including the Hippo-YAP signaling pathway, Hedgehog signaling pathway, Wnt/β-catenin pathway, E2F1-EZH2-SUZ12 and KMT1A-GATA3-STAT3 cascade (49, 55, 98–101). A recent single-cell sequencing study showed that variants of GPRC5A, MLL2, and ARID1A drive the proliferation of BCSCs (102). The revelation of the molecular mechanism of maintaining BCSCs is a very significant breakthrough in the therapeutic target of BC (92, 103).

### YAP induces and preserves stem-like qualities of BCSCs

Previous studies have shown that the Hippo-YAP pathway is essential to maintain the stem-like properties of some CSCs (41), such as BC, prostate cancer, breast cancer, lung cancer, and glioblastoma. YAP is a key regulatory protein for CSCs proliferation and carcinogenesis (55, 104–106). YAP is also of great significance in BCSCs. The research of Dr. Wang and Dr. Zhao shows that YAP is necessary for the proliferation and maintenance of stem-like properties of BCSCs and is related to its expressing OV6 and ALDH1 (49, 55).

**Figure 4:**
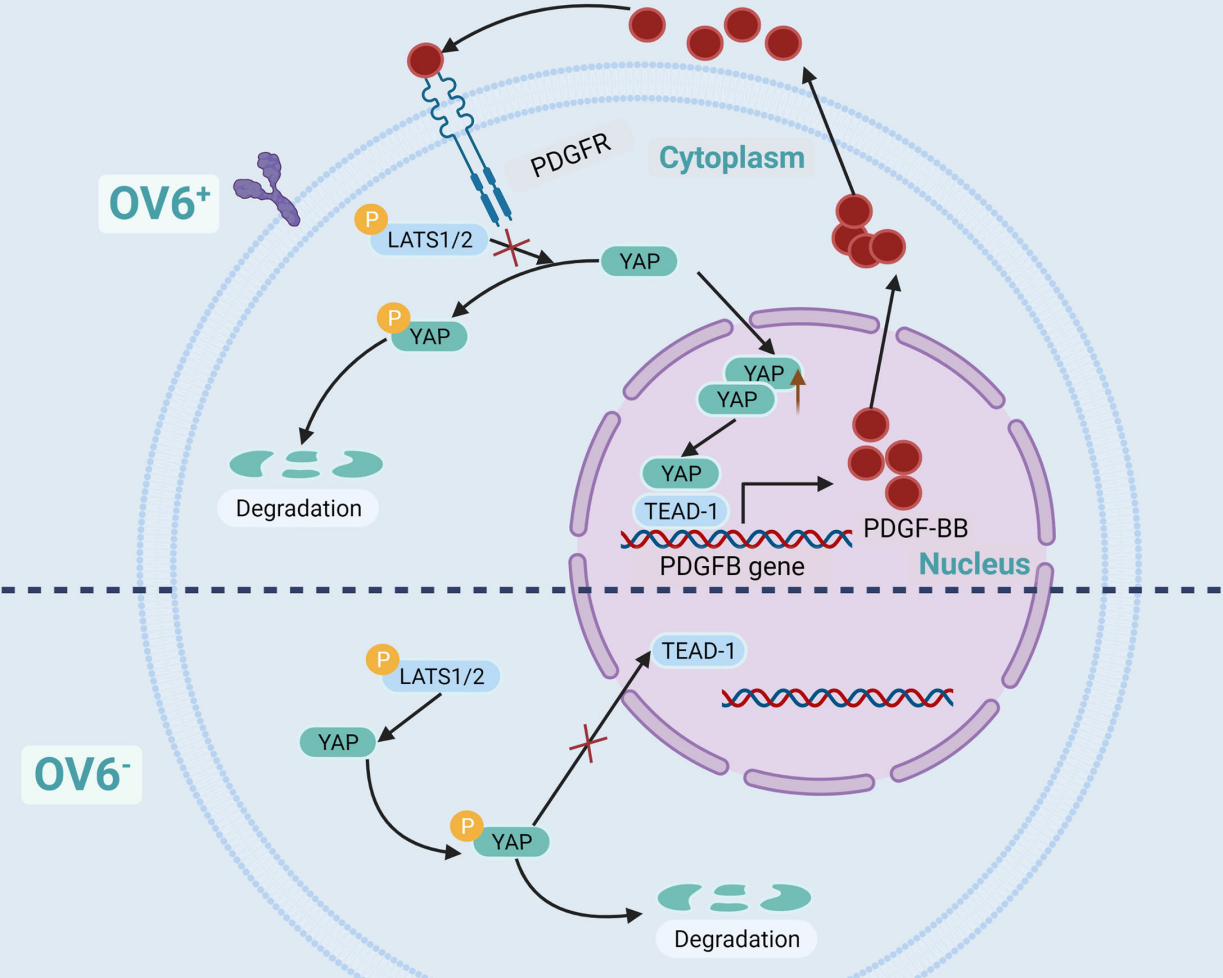
YAP/TEAD-1/PDGFBB/PDGFR positive feedback regulatory loop in OV6^+^ BCSCs.

OV6 is a unique marker of CSCs in epithelial malignant tumors, such as BC, hepatocellular carcinoma, cholangiocarcinoma, and esophageal cancer. CSCs are highly expressed and are associated with poor prognosis (55, 107–110). Dr. Wang et al. have found that BC cells in OV6^+^ have strong characteristics of tumor stem-like cells, which can significantly inhibit its proliferation and chemotherapy resistance when YAP is knocked out. Further experiments showed that YAP maintained the stem-like properties of BC cells of OV6^+^ by activating PDGFB, and the cells lost the characteristics of stem-like when PDGFB was knocked out. The use of YAP or PDGFR inhibitors in a mouse model of BC can block the positive feedback regulatory loop of BCSCs of OV6+, thereby overcoming the resistance of advanced BC to cisplatin (55). Dr. Wang’s research demonstrated that there is a positive feedback regulatory pathway in BC cells of OV6^+^. YAP activates PDGFB gene transcription and translation through TEAD-1 to produce PDGF-BB (Platelet-derived growth factor subunit B protein), which in turn prevents YAP from being phosphorylated by LATS1/2, thereby increasing the nuclear localization of YAP (55) (**Figure 4**).

YAP activity was also found in BCSCs cells of ALDH1+. When YAP was inhibited, the expression of ALDH1 decreased, it was more sensitive to chemotherapeutic drugs, and the ability of self-renewal and proliferation decreased significantly (49). In addition, it was also found that Hippo-YAP and COX2/PGE2 pathways co-acted on the proliferation of BCSCs, and their inhibitors successfully blocked the progression of BC (111). Moreover, YAP induces non-CSCs into CSCs (17) and maintains the characteristics of CSCs by inducing autophagy (112). These researches suggest that the Hippo-YAP pathway plays an important role in the proliferation and development of BCSCs and BC.

## The HIPPO-YAP in chemotherapy resistance and immunotherapy

### Mechanisms of chemotherapy resistance in BC

Drug resistance to chemotherapy and targeted chemotherapy remains a major obstacle to the treatment of various cancers, including BC (4, 113). The causes of chemotherapy resistance are very complex and can be divided into congenital resistance and secondary resistance according to their essential causes. Congenital resistance refers to mutations in the genome or epigenetic mutations that have occurred before treatment. Secondary resistance refers to genomic alterations that occur after treatment with the appropriate drug (113). Several prevalent mechanisms of drug resistance have been reported, such as increased drug efflux, drug target mutations, cell stemming, apoptotic escape, immune escape, and DNA damage repair (114–118). Among them, the role of cell stemness and apoptotic escape in chemotherapy resistance has been emphasized. The active DNA repair capacity and resistance to apoptosis that are characteristic of cell stemness are the main mechanisms of its resistance (119–121). Therefore, further studies targeting the mechanisms that maintain cell stemness are important to improve chemotherapeutic efficacy.

### The role of Hippo-YAP in chemotherapy resistance of BC

YAP is reported to be associated with drug resistance, such as cisplatin (122, 123), survivin and erlotinib inhibitors (124), anti-tubulin drugs (125), and radiation therapy (126). The sensitivity of cisplatin was negatively correlated with the expression of YAP in BC (127). Overexpression of YAP in BC was significantly correlated to resistance to cisplatin. Knocking out of the YAP gene not only increased the sensitivity of BC to cisplatin (50, 127) but also increased the sensitivity to other DNA damage drugs (50). YAP was recently reported to mediate chemotherapy resistance by maintaining tumor cell stemness (49, 55). Although there is a lot of evidence that YAP plays an important role in chemotherapy resistance of BC, the specific mechanism of YAP leading to chemotherapy resistance of BC is limited.

**Figure 5:**
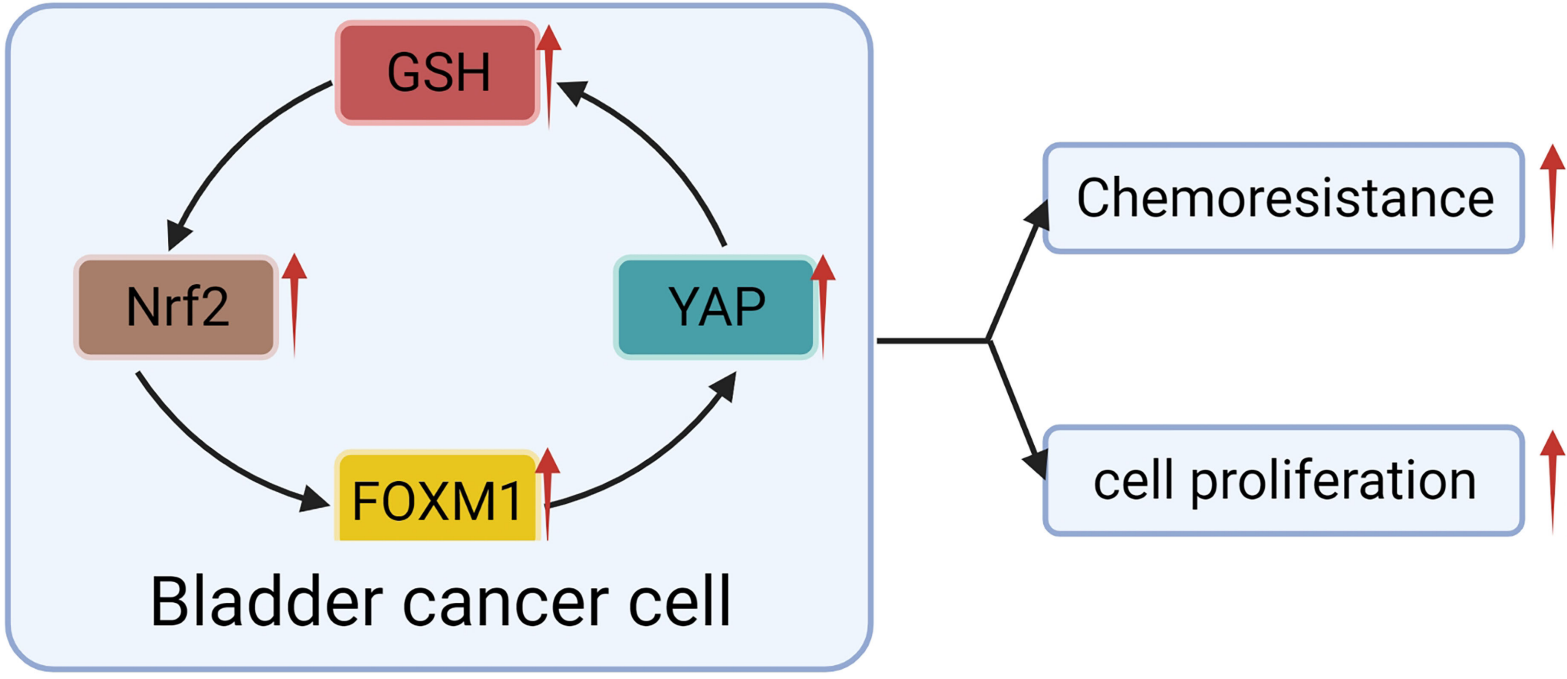
YAP crosstalk with Nrf2 leads to BC progression and chemotherapy resistance.

Fortunately, a recent study showed that YAP crosstalk with NRF2, thereby enhancing the antioxidant capacity of tumor cells that mediated BC chemotherapy resistance (50). The escape of apoptosis mediated by antioxidation is recognized as the mechanism of drug resistance in BC (50, 113). NRF2 is a classical regulator of cellular redox response (128, 129). With further research, it has been found that NRF2 has a specific high expression in cancer cells, can promote the progression (129) and metastasis (130) of many kinds of cancer, and make the human body resistant to chemotherapy and radiotherapy (131, 132). The interaction between NRF2 and YAP was found in BC cells. Knocking-out of NRF2 not only inhibited the proliferation and invasion of BC cells but also significantly restrained the expression of YAP (50). When YAP was blocked, the growth, invasion, and NRF2 expression of cancer cells were significantly decreased (50). For example, the chemotherapeutic drug-resistant cell lines were more responsive to Aila (YAP and NFR2 inhibitors) (133). Researchers suggested that NFR2 may interact with YAP through FOXM1 (50). A significant correlation was found among the expression of NFR2, FOXM1, YAP, and GSH in chemotherapy-resistant BC cell lines (50). When NFR2 was knocked out, the expression of YAP, FOXM1 and GSH decreased synchronously, along with decreased proliferation ability of the cell line and increased sensitivity to chemotherapeutic drugs (50). Although the evidence of direct interaction between NFR2 and FOXM1 is not sufficient but combined with the experiments of Dr. Gucci and Professor Eric Ciamporcero, we can speculate that there is a vague interaction between NFR2 and YAP in BC, which plays a role in regulating chemotherapy resistance of BC (**Figure 5**).

### Potential role of Hippo-YAP in immunotherapy

Immunotherapy has been widely demonstrated to be effective in BC and is currently a second-line treatment option for metastatic BC and a first-line treatment option for cisplatin-ineligible PD-L1+ patients (4, 5). However, the benefit of immunotherapy for BC patients is limited because of its complex tumor microenvironment-mediated immune escape and the low responsiveness of immunotherapy (5). Although no studies related to the Hippo pathway with immune escape and immunotherapy in BC. However, YAP was found to increase tumor immune escape response by increasing PD-L1 expression in other cancers, such as melanoma (134), and colorectal cancer (135). Interestingly, it was found that in lung cancer, YAP expression increased anti-tumor immune response by decreasing PD-L1 expression (136). Based on the available evidence the Hippo-YAP pathway has a quite complex role in tumor immunity with tissue heterogeneity. Therefore, revealing the role of Hippo-YAP in anti-tumor immunity in bladder cancer may be important for improving the efficacy of immunotherapy in the future.

## Clinical potential of HIPPO-YAP pathway for BC therapy

### The preclinical attempt targeted YAP-TEAD

The aberrant activation of YAP in BC leads to tumor recurrence and chemoresistance, which are major clinical difficulties of BC therapy. Targeting Hippo-YAP possesses the potential in solving this major obstacle. Since YAP exerts transcriptional activity primarily by binding to the transcription factor TEAD-1 (26, 27, 137, 138), inhibition of this interaction makes it the most direct and effective (138). Verteporfin (VP) inhibits the interaction of YAP with TEAD-1 by binding YAP (139). *In vitro* experiments demonstrate that VP inhibits BC growth and the stem-like properties of BCSCs (140–142). Although VP is used to treat macular degeneration, its low metabolic rate and low specificity *in vivo* make it toxic (143, 144), hindering its future use in cancer therapy. VGLL4 (Vestigial like family member 4) binds TEAD-1 competitively with YAP through the TDU (Tondu) structural domain, thereby reducing the transcriptional benefit of YAP (145, 146). Super-TDU (VGLL4-mimetic peptide) has significant anticancer effects in a mouse gastric cancer model induced by Helicobacter pylori (145). It has been reported that a YAP analog, namely 17-peptide (147, 148), has now been designed with a super-inhibitory effect on YAP-TEAD-1 and a significant inhibition of tumor proliferation in an ovarian cancer animal model (149). Unfortunately, even though breaking the YAP-TEAD-1 interaction is the most direct way to target the Hippo-YAP pathway, there are still no relevant drugs approved for clinical treatment of BC use.

### Activating Hippo kinase cascade would be a promising attempt

Hippo-kinase cascade, consisting mainly of the MST1/2, LAST1/2, and MAP4K families, whose activation inhibits the transcriptional function of YAP/TAZ (150). Thus, activation of the Hippo-kinase cascade is a viable way to target the Hippo-YAP pathway for cancer treatment. SHAP (STRN3-derived Hippo-activating peptide), a potent activator of MST1/2 enzymes, has better inhibitory effects on YAP than drugs such as VP and super-TDU, in addition to advantages toxicity and physical properties (151). In a mouse model of gastric cancer, SHAP exhibited stronger tumor-suppressive effects than drugs such as VP and super-TDU (151). The RAF (rapidly-accelerated fibrosarcoma) family was shown to inactivate MST1/2 by a mechanism acting upstream of the MST1/2 kinase (152). Therefore, inhibition of RAF leads to activation of MST1/2, which acts as an anticancer agent. Previously, ISIS-1532 oligonucleotide was found to silence the expression of RAF (153, 154). Although ISIS-1532 had a good response in lung cancer (153, 154), however, it performed poorly in phase II clinical trials in people with colon cancer, prostate cancer, and ovarian cancer (154–157). Despite the lack of studies on Hippo-kinase cascade activators in BC, this type of activator holds remarkably positive promise in the treatment of BC (144).

## Conclusion and perspective

Overexpression of YAP was verified, and current studies indicated that YAP has a more extensive contribution to the development of BC. YAP plays a key role in BC initiation, progression, chemoresistance, and induction of BCSCs (44, 45, 47, 55). Interestingly, multiple mechanisms are now found to be involved in YAP upregulation in BC. Therefore, the development of inhibitors of YAP is a promising direction. However, current molecular drugs faced a series of challenges, including insufficient clinical trials, uncontrolled side effects, metabolism difficulties, etc. Hence, drug metabolism and toxicology are urgent in the future development of YAP-related drugs. New drug design strategies, like antibody-drug coupling (ADC), should be a promising direction. Moreover, YAP-based chemicals are hard to compare favorably with traditional chemotherapy drugs in killing cancer cells frankly. However, they would more adjuvant drugs in overcoming the chemotherapy resistance than a single therapeutic target.

Although the mechanisms of Hippo-kinase cascade regulation in BC are poorly understood. However, according to recent findings, targeting the Hippo cascade may be more effective than interfering with the YAP-TEAD combination. In animal models of gastric cancer, SHAP was more efficacious than the conventional direct inhibitors of YAP (151). Therefore, we believe that activation of the Hippo-kinase cascade is a promising direction for the treatment of malignancies. However, developing protein activators is significantly more challenging than protein inhibitors. Therefore, further unraveling the mechanism of Hippo-kinase cascade dysregulation and developing related drugs are important for improving the clinical prognosis and developing individualized treatment plans for BC patients in the future.

## Review strategy and methods

The review strategy and inclusion criteria as listed below. The Major review strategy: a total of 41 publications were retrieved from Pubmed with the search terms Hippo/YAP and bladder cancer/urothelial carcinoma/transitional cell carcinomas, including 7 reviews and 34 research articles. The final selection of 22 articles (19 articles and 3 reviews) was based on the inclusion criteria (a. Subjects with bladder cancer or bladder cancer cell lines; b. independent cohort validation with relevant biomarker studies; c. Complete and appropriate controlled experiments). The minor review strategy: 1. Hippo/YAP and CSCs/cancer stem cells/bladder cancer stem cells 178(58 reviews and 120 papers); 2. Hippo/YAP and chemotherapy resistance/immunotherapy 82(22 reviews,60 papers); 3. Hippo/YAP and therapy 481 (151 reviews and 330 papers).

## Author contributions

GZ, JZ, and XC designed the thesis and outline for the review. LD and GX searched related publications. XC, KL, and RH drafted the manuscript. GZ, JZ, and XZ reviewed the manuscript and polished the grammar. All authors contributed to the manuscript revision and approved the submitted version.

## Funding

This work was supported by the National Natural Science Foundation of China (No. 81860456 and 81760462); Training plan for the academic and technical leaders of major subjects in Jiangxi Province (No. 20213BCJL22038); Science and Technology Research Project of Jiangxi Provincial Education Department (GJJ211550 and GJJ211523).

## Conflict of interest

The authors declare that the research was conducted in the absence of any commercial or financial relationships that could be construed as a potential conflict of interest.

## Publisher’s note

All claims expressed in this article are solely those of the authors and do not necessarily represent those of their affiliated organizations, or those of the publisher, the editors and the reviewers. Any product that may be evaluated in this article, or claim that may be made by its manufacturer, is not guaranteed or endorsed by the publisher.

## Abbreviations

ABCG2, ATP-binding cassette super-family G member 2; ADC, antibody-drug coupling; ALDH1, Aldehyde dehydrogenase 1 family, member A1; AMPK 5’, AMP-activated protein kinase; ANKRD1, Ankyrin repeat domain-containing protein 1; ARID1A, AT-rich interactive domain-containing protein 1A; BC, Bladder cancer ; BCSCs, bladder cancer stem-like cells ; BMI1, Polycomb complex protein BMI-1; CD133, antigen; CD44, CD44 antigen; COX2, prostaglandin-endoperoxide synthase 2; CSCs, cancer stem cells ; CTGF, connective tissue growth factor; CYR61, Cysteine-rich angiogenic inducer 61; DUB, de-ubiquitin enzyme; E3, ubiquitin-protein ligases; FBXW7, F-box/WD repeat-containing protein 7; FOXM1, Forkhead box protein M1; GNA11, Guanine nucleotide-binding protein subunit alpha-11; GNA12, Guanine nucleotide-binding protein subunit alpha-12; GNA13, Guanine nucleotide-binding protein subunit alpha-13; GNAI2, Guanine nucleotide-binding protein G(i), alpha-2 subunit; GNAQ, Guanine nucleotide-binding protein G(q) subunit alpha ; GNAS, Heterotrimeric G-protein alpha subunit Gs-α; GPCRs, G-protein-coupled receptors; GPRC5A, Retinoic acid-induced protein 3; GSH, Glutathione; ITCH, itchy E3 ubiquitin protein ligase; LATS1, Large tumor suppressor kinase 1 ; LATS2, Large tumor suppressor kinase 2; MAP4K, Mitogen-activated protein kinase kinase kinase kinase; MIBC, muscle-invasive bladder cancer; MINDY1, MINDY lysine 48 deubiquitinase 1; MLL2, Histone-lysine N-methyltransferase 2D; MST1, macrophage-stimulating 1; MST2, Serine/threonine-protein kinase 3; NMIBC, nonmuscle-invasive bladder cancer; NRF2, Nuclear factor erythroid 2-related factor 2; NUAK2, NUAK family SNF1-like kinase 2; OV6, Ov6 protein; PDGFB, Platelet-derived growth factor subunit B; PDGF-BB, Platelet-derived growth factor subunit B protein; PD-L1, Programmed cell death 1 ligand 1; PGE2, Prostaglandin E2; PRAJA1, E3 ubiquitin-protein ligase Praja1 ; RAF, rapidly-accelerated fibrosarcoma; RASSF1, Ras association domain-containing protein 1; Rho, GTPase Rho family of GTPases; RhoGEF, RhoGEF domain; RhoA, Ras homolog family member A; RhoB, Ras homolog family member B; RhoC, Ras homolog family member C; SHAP, STRN3-derived Hippo-activating peptide; SIAH2, siah E3 ubiquitin protein ligase 2; Super-TDU, VGLL4-mimetic peptide; TAZ, Tafazzin; TEAD-1, TEA domain family member 1; TURBT, transurethral resection of bladder tumor; UPS, ubiquitin proteasomes system ; VGLL4, Vestigial like family member 4 ; VP, Verteporfin; WWP1, WW domain containing E3 ubiquitin protein ligase 1; YAP, Yes-associated protein 1; 17-peptide, YAP-like peptide

## Glossary

ABCG2: ATP-binding cassette super-family G member 2
ADC: antibody-drug coupling
ALDH1: Aldehyde dehydrogenase 1 family, member A1
AMPK 5’: AMP-activated protein kinase
ANKRD1: Ankyrin repeat domain-containing protein 1
ARID1A: AT-rich interactive domain-containing protein 1A
BC: Bladder cancer
BCSCs: bladder cancer stem-like cells
BMI1: Polycomb complex protein BMI-1
CD133: antigen
CD44: CD44 antigen
COX2: prostaglandin-endoperoxide synthase 2
CSCs: cancer stem cells
CTGF: connective tissue growth factor
CYR61: Cysteine-rich angiogenic inducer 61
DUB: de-ubiquitin enzyme
E3: ubiquitin-protein ligases
FBXW7: F-box/WD repeat-containing protein 7
FOXM1: Forkhead box protein M1
GNA11: Guanine nucleotide-binding protein subunit alpha-11
GNA12: Guanine nucleotide-binding protein subunit alpha-12
GNA13: Guanine nucleotide-binding protein subunit alpha-13
GNAI2: Guanine nucleotide-binding protein G(i), alpha-2 subunit
GNAQ: Guanine nucleotide-binding protein G(q) subunit alpha
GNAS: Heterotrimeric G-protein alpha subunit Gs-α
GPCRs: G-protein-coupled receptors
GPRC5A: Retinoic acid-induced protein 3
GSH: Glutathione
ITCH: itchy E3 ubiquitin protein ligase
LATS1: Large tumor suppressor kinase 1
LATS2: Large tumor suppressor kinase 2
MAP4K: Mitogen-activated protein kinase kinase kinase kinase
MIBC: muscle-invasive bladder cancer
MINDY1: MINDY lysine 48 deubiquitinase 1
MLL2: Histone-lysine N-methyltransferase 2D
MST1: macrophage-stimulating 1
MST2: Serine/threonine-protein kinase 3
NMIBC: nonmuscle-invasive bladder cancer
NRF2: Nuclear factor erythroid 2-related factor 2
NUAK2: NUAK family SNF1-like kinase 2
OV6: Ov6 protein
PDGFB: Platelet-derived growth factor subunit B
PDGF-BB: Platelet-derived growth factor subunit B protein
PD-L1: Programmed cell death 1 ligand 1
PGE2: Prostaglandin E2
PRAJA1: E3 ubiquitin-protein ligase Praja1
RAF: rapidly-accelerated fibrosarcoma
RASSF1: Ras association domain-containing protein 1
Rho: GTPase Rho family of GTPases
RhoGEF: RhoGEF domain
RhoA: Ras homolog family member A
RhoB: Ras homolog family member B
RhoC: Ras homolog family member C
SHAP: STRN3-derived Hippo-activating peptide
SIAH2: siah E3 ubiquitin protein ligase 2
Super-TDU: VGLL4-mimetic peptide
TAZ: Tafazzin
TEAD-1: TEA domain family member 1
TURBT: transurethral resection of bladder tumor
UPS: ubiquitin proteasomes system
VGLL4: Vestigial like family member 4
VP: Verteporfin
WWP1: WW domain containing E3 ubiquitin protein ligase 1
YAP: Yes-associated protein 1
17-peptide: YAP-like peptide
